# Supplementary material for: Identification of photoperiod-induced specific miRNAs in the adrenal glands of Sunite sheep (Ovis aries)
Source: Front Vet Sci. 2022 Jul 22;9:888207. doi: 10.3389/fvets.2022.888207 (PMC9354845; doi:10.3389/fvets.2022.888207)
Supplement: Supplementary file 1 [file Data_Sheet_1.ZIP › Supplementary file/miRDeep2 file.zip!/miRDeep2 file/Know_Structure/SPLP42A3/oar-let-7d.pdf]

miRBase precursor	: oar-let-7d
Total read count	: 12986
oar-let-7d read count	: 12020
remaining reads	: 966

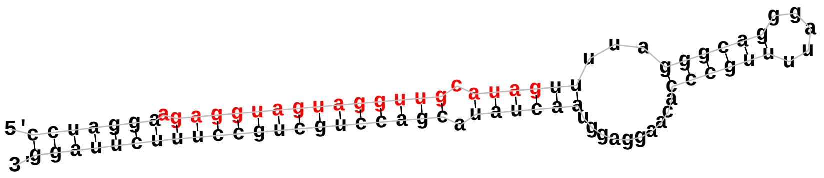

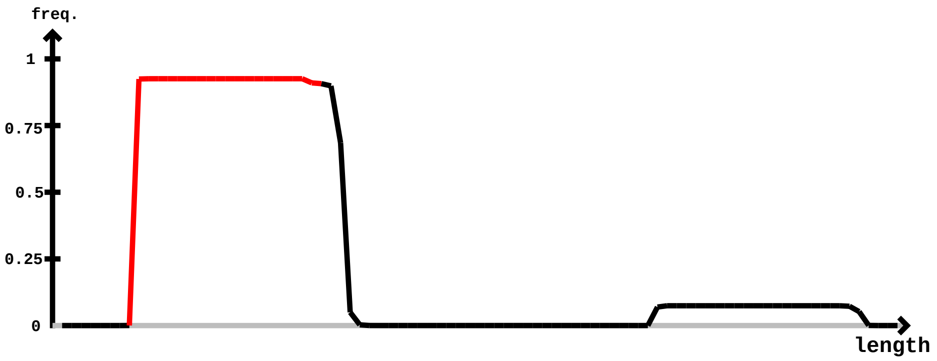


```
          oar-let-7d
5'- ccuaggaagagguaguagguugcauaguuuuagggcagggauuuugcccacaaggagguaacuauacgaccugcugccuuucuuagg   -3'    exp
    ((((((.(((((((((((((((.((((((...((((((.....))))))..........)))))).)))))))))))))))))))))   reads      mm         sample
    ......aagagguaguagguugcauag............................................................   1          0          seq
    ......aagagguaguagguugcauagu...........................................................   2          0          seq
    ......aagagguaguagguugcauaguu..........................................................   1          0          seq
    .......agagguaguagguugcau..............................................................   202        0          seq
    .......agagguaguagguuAcau..............................................................   1          1          seq
    .......agagguaguagguugcaua.............................................................   38         0          seq
    .......agagguaguagguugcauag............................................................   108        0          seq
    .......agagguaguagguugcauagu...........................................................   2770       0          seq
    .......agagguaguagguugcauaguu..........................................................   8240       0          seq
    .......agagguaguagguugcauaguuu.........................................................   606        0          seq
    .......agagguaguagguugcauaguuuu........................................................   36         0          seq
    .......agagguaguagguugcauaguuuua.......................................................   1          0          seq
    ........gagguaguagguugcauagu...........................................................   3          0          seq
    ........gagguaguagguugcauaguu..........................................................   9          0          seq
    ........gagguaguagguugcauaguuu.........................................................   1          0          seq
    .........agguaguagguugcauagu...........................................................   1          0          seq
    .............................uuagggcagggauuuugcccacaaggaggu............................   1          0          seq
    ............................................................acuauacgaccugcugccuuucu....   1          0          seq
    .............................................................cuauacgaccugcugccuu.......   2          0          seq
    .............................................................cuauacgaccugcugccuuu......   22         0          seq
    .............................................................cuauacgaccugcugccuuuc.....   248        0          seq
    .............................................................cuauacgaccugcugccuuucu....   626        0          seq
    .............................................................cuauacgaccugcugccuuucuu...   2          0          seq
    ..............................................................uauacgaccugcugccuuu......   1          0          seq
    ..............................................................uauacgaccugcugccuuuc.....   11         0          seq
    ..............................................................uauacgaccugcugccuuucu....   47         0          seq
    ..............................................................uauacgaccugcugccuuucuu...   5          0          seq
```
